# On the Present State of Medicine in Prussia

**Published:** 1836-10

**Authors:** J. F. C. Hecker

**Affiliations:** Professor of Medicine in the University of Berlin


### PART FOURTH.

### Medical Intelligence.

---

### ON THE PRESENT STATE OF MEDICINE IN PRUSSIA;

By J. F. C. Hecker, M.D., Professor of Medicine in the University of Berlin.

### SECOND REPORT.

### MEDICAL SOCIETIES IN PRUSSIA.

The great general object of Medical Societies is to promote Medical Science, by exciting their members to institute important enquiries, and to make well-grounded observations. All other points which claim attention in such societies,—as, for instance, the social union of the physicians of large towns, the communication of novelties in practice, and the reporting and discussion of medical cases,—are of a subordinate character, although they may be of themselves not undeserving of regard. In the present Report, those Medical Societies can alone claim our attention which stedfastly keep in view the great object of their institution, the promotion of science. Societies which only pursue common-place objects, in which mere form and personal interests are the most conspicuous features, are not at all adapted to excite a useful spirit in their members: indeed, oftentimes they only serve to promote partial views, and, as far as real science is concerned, their operation is perfectly futile.

In the present state of society, the establishment and maintenance of medical associations calculated to promote the progress of science in its higher import, is certainly attended with considerable difficulty. The intellectual combination of the people among themselves is more active than ever; literature has become more generally accessible, and the means of acquiring scientific knowledge have so extraordinarily increased on all sides, that it becomes necessary that all literary associations should possess members alike talented and active, in order that they may obtain and communicate a knowledge of all that is passing in the scientific world, and be able to promote and guide investigations amongst themselves. Thus it can easily occur (and examples of this kind are not unfrequent,) that an active and zealous editor of a Journal, assisted by talented correspondents, can effect more real good than academies whose forms and regulations are become obsolete.

No societies are more calculated, from the nature of their object, to widen and extend real science, than those constituted by the Medical Faculties of Universities. The experience of centuries has shown that they have effected the utmost good for the healing art: the efficiency and influence of professors are far from being confined within the narrow limits prescribed by the formal teaching of their particular branch, but extend, or ought to extend, to the higher and more general departments of science. To deny this, is to fail to comprehend the real spirit of universities; and, should ever this crude idea (which we hear sometimes expressed,) gain ground, we shall most certainly find that the higher scientific branches of medicine will perish in the universities, and the mere practical and manual parts of medical and surgical study will receive attention. Medical Faculties, however, cannot, from their nature and duties, answer all the objects which are desirable; so that the existence of Medical Associations, in addition to them, is not only useful but necessary. Such associations do much good, by promoting the composition and publication of monographs on difficult subjects, through the institution of prize essays, as well as by exciting general zeal amongst their members. It has frequently happened that learned societies of this kind have given a decided and advantageous direction to medical science, as in the case of the Medical and Chirurgical Society of London, the Académie de Médecine de Paris, and many others.

### I. THE ROYAL ACADEMY OF THE SCIENCES.

*(Die Königliche Akademie der Wissenschaften.)*

This is the most celebrated and important of the learned societies in Prussia. It was established in the year 1700, by Frederick the First, after the plans and arrangements of that celebrated philosopher, Leibnitz, who was also its first president. Since that time this Society has undergone many changes, amongst the most important of which was that which took place under Frederick the Great. He, having invited to his court a considerable number of celebrated French men of learning, the whole Academy assumed a French complexion, and even its Memoirs were published in the French language. This degrading practice, however, has been long since discontinued, and the Transactions of the Society, which may vie with those of the best academies in Europe in solidity and importance, appear again in the German language. The Academy consists at present of four classes,—the Physical, Mathematical, Philosophical, and Historico-philological. It is proposed to unite these four classes into two groups, namely, Physico-mathematical and Philosophico-historical: however, nothing is as yet decided respecting this alteration. At present the first two classes, as well as the two last, have only one secretary.

The Academy has no president. The king is protector, and confirms the appointment of all new members, whether elected by the members or proposed by the minister of instruction, *(minister der unterrichts angelegenheiten.)* The members are Ordinary, (those who reside in Berlin,) Foreign, and Honorary: among the last are included a considerable number of corresponding members. The Academy has its appropriate building and library, which last principally consists of the Transactions of the most important learned Societies: its income is inconsiderable, and is disposed of partly in salaries to most of the ordinary members, partly in defraying the expense of the publication of its Transactions, and partly as premiums for prize dissertations.

Among the physicians and naturalists who are members of the Academy are Alexander von Humboldt, Leopold von Buch, Erman, Link, Mitscherlich, Ehrenberg, Horkel, Klug, Mueller, Kunth, &c. These belong to the physical class, and their valuable labours are directed to the various branches of the natural sciences. From Ehrenberg and Mueller alone we receive sometimes physiological communications of distinguished value. Practical medicine, in the strict sense of the term, and all that stands in immediate relation to it, is excluded from the higher departments of the Academy.

### II. THE IMPERIAL LEOPOLD AND CHARLES'S ACADEMY OF NATURALISTS.

*(Academia Cæsarea Leopoldino-Carolina Naturæ curiosorum.)*

This institution was not originally of Prussian origin, although it at present flourishes in the Prussian dominions. It possesses the peculiar and very liberal feature that it is not attached to any particular state, but can choose its president from any of the German territories; and the president so chosen can obtain possession of the archives and library of the Academy, whether he lives in Prussia, Austria, Bavaria, or Saxony.

This Academy is one of the oldest in Germany: it was founded by Johann Lorenz Bausch, a physician in Schweinfurt Franconia. In 1651, he circulated an invitation to all the learned physicians in Germany, to communicate to him any important and rare observations. This proposal gave such satisfaction that, in the following year, 1652, a meeting of physicians took place at Schweinfurt, when Bausch was elected president. The regulations of the

Society, however, were not drawn up until after the death of Bausch, in 1665, when his successor, Johann Michael Fehr, (likewise a physician of Schweinfurt,) completed them. The first collection of the Academy's observations was completed in 1760, but was not published until 1684, under the title "Miscellanea curiosa, sive Ephemeridum Medico-physicarum Germanicarum Academiæ naturæ curiosorum, Decuriæ I. Annus I. Anni 1670, continens celeberrimorum medicorum in et extra Germaniam observationes medicas et physicas, vel anatomicas, vel botanicas, vel pathologicas, vel chirurgicas, vel therapeuticas, vel chymicas." *Frankofurti et Lipsiæ*, 1684, 4to. This, the first volume, was followed by a number of others, but with considerable interruptions. The whole series serves to show, in a manner in every respect interesting, the spirit and progress of the natural sciences in Germany, and indeed has contributed a not unimportant part to the general advancement of science. In the year 1687, the Emperor Leopold I. granted to the society many important privileges, which were still farther extended by Charles VI. (1711-1740). From these two emperors it takes its present name, and it had to thank them for the means of extending its influence. The valuable library of the academy was first founded about 1730, by the then President, *Baier*, a Professor of Medicine in Altorf; and was afterwards increased by important presents. It is at present preserved in Bonn, although the president, Professor Nees von Esenbeck, lives in Breslau. It is to be attributed to the influence of this celebrated president that the academy applies itself principally to the natural sciences, and does very little for the promotion of pure medical science, as the last vol. "Nova Acta Academiæ, etc." *Bonn* and *Breslau* 1835, 4to., will easily prove.

The academy, however, can boast of possessing the labours of the most distinguished men of the day among its collection, not alone Germans, but also many foreigners. The number of the members who have belonged to the Academy since its commencement is 1,324. At present there are no general meetings of the members, but the business of the society is transacted by letter; and in Prussia free communication is favoured by the allowance of letters on the business of the Society going through the post free of charge. Even down to the present time the antique custom is preserved, by which every member receives a by-name in addition to his own proper name, which additional name must have some historical relation to its bearer: for instance, the renowned Clot-Bey is surnamed *Oribasius,* Professor Pommer of Zurich is known as *Petit,* and the present President is styled *Aristoteles*.

The foregoing are, properly speaking, scientific and not medical Societies; there exist, however, many strictly of this class in the various towns. I will notice the most important.

### III. HUFELAND'S MEDICO-CHIRURGICAL SOCIETY OF BERLIN.

(*Die Hufelandische Medicinisch-chirurgische Gezellschaft in Berlin.*)

This Society was founded in 1810, by Christoph Wilhelm Hufeland, and has borne his name since 1833. Its object is the practical promotion of Medicine and Surgery, and, at the same time, the establishment of a centre for Collegiate Union in Berlin. Therefore, it dispenses as much as possible with all outward formalities, banishing constraint and ceremony, which in the end make these societies often mere parade and extinguish their invigorating spirit. Its meetings are characterised by friendly communication and cordial cooperation.

The Society contains none but active members, and their number is not limited. Since its object is not so much of a public as private character, being rather *inward* than *outward,* it has no honorary members: still its rules provide for corresponding members, for the reciprocal communication of statistical accounts of weather and general medical practice, information as to the prevalence of epidemic or contagious diseases, new remedies, and points of practice, and all new discoveries and accidents relating to medicine.

A committee of ten members is chosen annually, whose business it is to provide for the general welfare of the Society, to elect new members, to propose new laws and regulations, which, however, must be sanctioned by a majority of a general meeting, before they can come into operation. From among these ten is chosen a director, who holds the situation during his life: it is his duty to open and close the meetings of the Society; to arrange the days of meeting throughout the year, and prepare the list of subjects to be brought before the meeting, with the names of the contributing members; to issue notices to the members, to report on recent occurrences, to introduce strangers to the Society, &c. Two Secretaries are also elected, one for the internal arrangement and the other for the foreign business of the Society. The first has an assistant-secretary, and the other two. The appointment to these posts lasts two years, and can be renewed at the end of that period.

The Secretary for the internal department is obliged to be present at every meeting, to keep the minutes of the Society; or, when he is prevented attending, his deputy occupies his place.

The Secretary for external business is bound to conduct the foreign correspondence, and to preserve reports of the various communications. A Censor is likewise chosen every year, to preserve due order in the meetings. The Librarian, besides the care of the library, superintends the circulation of journals, &c. and the finance department.

New members can be elected every three months, in the following manner: 1, the proposed member must be announced to the director; 2, the director proposes the candidate to the committee, who decide upon his admission; 3, if elected, he is introduced to the Society; but, 4, if rejected, the strictest secrecy is maintained.

The Society holds its meetings every fortnight, on Friday, at five o'clock; the business commences at a quarter past, and is conducted in the following manner: 1st, The reports of the general state of health, the barometrical and thermometrical registers, the prevailing diseases and mortality of the last fortnight, are read by the Secretary. 2, A lecture, or communication of some kind, is made by the member whose name stands on the list for the evening; 3, discussion and observations from the other members. Every physician who is a stranger in Berlin can be introduced to the meetings by a member; but he must be either first announced to the director, or else introduced to him at the time of assembling, and be then, by him, introduced to the meeting.

Berlin physicians, who are not members of the Society, and who wish to be present at any of its meetings, must be announced to the Society on a previous evening through one of the members. The anniversary of the Society, the 1st February, is commemorated by its members,—and, besides this, three other anniversaries are kept: viz. Harvey's, the 1st August; Haller's, the 1st November; and Jenner's, the 14th May. The Society's library consists principally of the best domestic and foreign journals, which previously circulate amongst the members.

The yearly subscription is four dollars (about twelve shillings,) for the general expenses of the Society, and each member is moreover bound to present a copy of any work he may publish to the Society. Since the time of its establishment, the *Hufelandische Gesellschaft* has always continued to fulfil the object of its founder in an exemplary manner; many papers have been brought before the Society possessing a real scientific value over and above their passing interests: it is therefore to be regretted that the Society has not published from time to time reports of at least the best of its fruits. It has, however, preferred a more modest retirement, choosing rather to confine its good effects to its own immediate circle, that extend its influence more remotely. Its transactions are therefore scattered among different journals, as separate essays, or they are entirely lost to the world. It may not be amiss to remark here, that the best form under which the works of really scientific medical societies can be published is beyond alldoubt that of the London Medico-Chirurgical Transactions; for, in such alone can the detailed monographical character of a scientific subject be done justice to: strict, precise, and many-sided investigations are the indispensable requisites of a monograph. Every other lighter form allows a too easy admittance of less important subjects. Writings of this character supersede the so-called "interesting cases," which are really interesting to none but their author, and such brief notices and novelties as can be got up by any physician, however little accomplished, with very little trouble, and which are forgotten in as short a time as was required for their production. Journals appearing at brief intervals, are unworthy of a respectable society; for, although, by their rapid publication, they may have some unimportant advantages, they are in reality mere concessions made to the superficiality of the mass of practical physicians, who have long given up all earnest study, and will only be instructed by such tea-and-coffee reading as will enable them to make it the subject of common conversation.

### IV. THE SILESIAN SOCIETY FOR NATIVE IMPROVEMENT AT BRESLAU.
### (*Die Schlesische Gesellschaft für Vaterländische Cultur in Breslau.*)

This Society has already existed a long time, and is laid out on a somewhat extensive scale; since it consists of the following departments: 1, for the study of the natural sciences; 2, botanical; 3, entomological; 4, geognostical (merely local, however;) 5, medical; 6, economical; 7, pedagogical; 8, historical; 9, technical; and 10, medical.

The medical section is of itself a complete and well-organized society: it consists of resident and provincial members, who all belong to the province of Silesia, also honorary and corresponding members. The Society meets every fortnight, and at its meetings are brought forward papers upon all medical sub jects, which give occasion to interesting discussions, since Breslau has the good fortune to possess many talented physicians. In 1829, the Society published its first collection of these treatises under the title, "Neue Breslauer Sammlungen aus der Gebiete der Heilkunde, Bd. I." (*New Breslau Collections in the department of Medicine*, Vol. I.) Since which, however, as far as I am aware, it has not been continued. This volume recalls to mind an old periodical journal, likewise published by a Society, and even so named, "die Breslauer Sammlungen," which appeared in the beginning of the last century, and, from the then great scarcity of periodicals, was much read, and, without doubt, effected much good. The Society also publishes every year an account of their transactions and changes: the last, dated Breslau, 1835, proves the praiseworthy activity of the Society. The general secretary of the Society is Professor Wendt, of Breslau. The secretary of the medical section, Dr. Borkheim.

### V. THE MEDICAL SOCIETY AT MUNSTER.
### (*Die ärztliche Gesellschaft zu Münster.*)

Notwithstanding Münster, as being the principal town of Westphalia, offers many circumstances favorable to the union of its physicians in general scientific pursuits, still it was not until 1827 that such a Society was firmly established; all earlier attempts at the completion of such a desirable object having completely miscarried. In the year named, a number of physicians and other scientific men combined under the above title; rules were drawn up, and approved; and from that time the Society has given, in its regular monthly meetings, full proof of its activity. At each of these meetings, a lecture is delivered, followed by short communications and discussions. A collection of the proceedings of the Society was published in 1829, under the title "*Abhandlungen und Beobactungen der Arztlichen Gesellschaft zu Münster*;" but, as far as I am aware, no continuation of them has appeared since that time. This, however, is not to be attributed to a diminished activity on the part of the Society, but arises from the fact of treatises and collections of this sort meeting with little encouragement in their sale; for, however sterling and important in a scientific point of view they may be, still they never make a profitable "market commodity;" whence it happens that most prefer inserting their treatises in the journals of the day, whereby they are sure to receive a more extended circulation.

### VI. THE SOCIETY FOR NATURAL AND MEDICAL SCIENCE AT BERLIN.
*(Die Gesellschaft für Natur-und Heilkunde in Berlin.)*

This Society was founded in 1810, having for its object the closer union of physicians who wished, through reciprocal communications, to extend the benefit of their scientific investigations. The pursuits of the Society are not limited to medicine in the narrower sense of the word, but include also the various branches of the natural sciences. Its meetings take place every month, when a lecture is delivered by one of the members. The Proceedings of this Society deserve the highest character for the spirit which has always distinguished them. It is therefore the more to be regretted that the Society has not published its labours, but has left it to the individual members either to publish their treatises in any of the journals of the day, or else withdraw them from the public entirely.

The late Dr. Heim, who died in 1834, was the former president of the Society, and the celebrated Rudolphi was the secretary. At present Link occupies the president's chair, and Ehrenberg (who, through his extraordinary talent for observation, has led to such important discoveries in Physiology, Comparative Anatomy, and Zoology,) is its present secretary.

### VII. THE MEDICAL ASSOCIATION OF PRUSSIA IN BERLIN.
*(Die Verein für Heilkunde in Preussen.)*

This Society was established 1832, in consequence of the want felt by many physicians of a sterling and well-edited journal, in which they might publish their observations. Regulations were immediately proposed, and the object of the Society was announced to be the promotion of scientific enquiry in the general circle of medical study. To obtain these ends, the following means were decided upon: 1st. The monthly meeting of the Society; and, 2dly, the publication of a weekly journal, under the title of the "*Medicinische Zeitung.*"

The Society consists, 1, of a Presidency, composed of the President, (the present is Dr. Rust,) Vice-President, Secretary, and Vice Secretary, and the Editor and Co-Editor of the Journal; 2, of a Committee for conducting the publication of the Journal; 3, of ordinary, honorary, and corresponding members. The ordinary members must be Prussian physicians, without reference to their place of residence in the Monarchy, and must have distinguished themselves not only by their general medical talents during many years' active exertion in practice, but must have proved their fitness as members through their general exertions in science, &c. In the meetings of the Society, the principal object is the promoting of reciprocal communications and more intimate acquaintance of the members: therefore, to accomplish this, no written lectures are allowed; but any remarks and results of the passing events, that possess medical or scientific importance, are made the subject of extemporary observation and general discussion. The newest productions of general literature, according to their subject, are also laid before the Society, with short remarks as to their value.

None but foreign physicians can be introduced to the meetings of the Society. The Society celebrates its anniversary on the day of its original establishment by a dinner, to which any distinguished strangers can be introduced, provided they have been previously made known to the presidency. In the course of the fortnight previous to the general annual meeting, the secretary lays before the Society the report of its proceedings, efficiency, and economical relations. At this meeting alone can any changes or extensions of the laws take place, as well as the selections of new members.

The Editor takes upon himself the charge of the editorship of the "*Zeitung,*" (the present Editor is Dr. Grossheim, who succeeded the writer of the present article in the beginning of this year,) in which he is assisted by the committee already spoken of.

The "Zeitung" includes all branches of medicine, but principally it contains articles on the following subjects:—

1. Reports on the efficiency and result of the various cliniques of the Prussian states.
2. The scientific results of the practice of Prussian physicians.
3. Anatomical, physiological, and pathological notices.
4. Report of the prevailing diseases both of men and cattle.
5. Remarks on new points of practice, or new remedies.
6. Cases from the department of state medicine.
7. Medical topography and statistics of Prussia.
8. Information on mineral springs and baths.
9. Meteorological reports and influence of weather on general health.
10. Notices of such legislative arrangements as are of importance in a medical point of view.
11. Personal notices of Prussian physicians; appointments, promotions, deaths, &c.
12. Bibliographical notices of native and foreign literature.

The articles of this journal which refer entirely to medical affairs, must be as short as possible, in order to maintain the character of the journal: voluminous dissertations are therefore entirely excluded.

Since the Journal belongs to no party, especial care is taken to exclude all personal polemics.

Every member is considered to be *ex officio* an Editor of the journal, and, as such, is expected in his turn to furnish articles for it, but with especial reference to such of its members as belong to clinical institutions; and those physicians of districts (who are appointed as such, and paid by the government in Prussia,) are desired to furnish such statistical reports of the eruption of epidemics, effects of vaccination, cases of midwifery, &c. as may appear of sufficient interest to merit a place in the journal.

